# Positivity analysis of *Bordetella* spp. and SARS-CoV-2 coinfections in clinical samples from Colombia, 2021–2022

**DOI:** 10.1128/spectrum.01242-25

**Published:** 2025-09-12

**Authors:** Valentina Bonilla-Bravo, Efrain Montilla-Escudero, Ximena Castro Martinez, Fabiola Rojas, Carolina Duarte, Sandra Aparicio, Sergio Yebrail Gómez-Rangel, Paula Rodriguez Romero, Sandra Lucero Bonilla, Franklyn Prieto Alvarado, Jaid Rojas Sotelo, Gloria Rey Benito

**Affiliations:** 1 Pan American Health Organization 119925 , Bogotá, Colombia; 2 Grupo de Microbiología, Subdirección Laboratorio Nacional de Referencia, Dirección de Redes en Salud Pública, Instituto Nacional de Salud 67626, Bogotá, Colombia; 3 Grupo de Transmisibles, Dirección de Vigilancia y Análisis de Riesgo en Salud Pública, Instituto Nacional de Salud 67626, Bogotá, Colombia; 4 Grupo de Virología, Dirección de Redes en Salud Pública, Instituto Nacional de Salud 67626, Bogotá, Colombia; 5 Pan American Health Organization 50514 , Washington, DC, USA; Yan'an University, Yan'an, China

**Keywords:** coinfection, *Bordetella *spp., SARS-CoV-2, pertussis surveillance

## Abstract

Pertussis and COVID-19 are respiratory diseases with similar clinical manifestations, complicating diagnosis. In Colombia, pertussis surveillance is based on probable cases confirmed through laboratory detection of *Bordetella pertussis*. During the COVID-19 pandemic, pertussis incidence declined sharply. Given limited data on coinfections with COVID-19 and pertussis, this study aimed to estimate the positivity of *Bordetella* spp. in SARS-CoV-2-positive samples collected in Colombia during 2021–2022. A retrospective analysis was conducted on samples collected between January 2021 and December 2022 through simple random sampling from specimens stored at the National Reference Laboratory (NRL). Demographic data, symptoms, complications, and race were obtained from the National Surveillance System (SIVIGILA), and PCR results were used to identify *Bordetella* spp. coinfections. National and departmental positivity rates were estimated with Clopper-Pearson confidence intervals. PCR testing for *Bordetella* spp. was positive in 0.54% (6/1,102) of samples, with four cases involving B. pertussis and two involving *B. parapertussis*; no *B. holmesii* coinfections were detected. The national positivity rate of SARS-CoV-2 and *Bordetella* spp. coinfections was 1.92% in 2021, decreasing to 1.25% in 2022. Although coinfections with viral agents and *B. pertussis* are common, this study found low positivity of SARS-CoV-2 and *Bordetella* spp. coinfections in Colombia during 2021–2022, likely due to non-pharmacological interventions, the cyclical pattern of *B. pertussis*, and possible underestimation related to the geographic and demographic scope of the sample. This is the first report of such coinfections in Colombia, offering a reference for future studies in Latin America.

**IMPORTANCE:**

This is the first report of SARS-CoV-2 and *Bordetella* spp. coinfections in Colombia, providing key evidence of their low occurrence during 2021–2022. Given that both diseases share clinical features and may complicate differential diagnosis, these findings underscore the importance of integrated surveillance for respiratory pathogens. The observed decrease in positivity may be linked to non-pharmaceutical interventions and the cyclical nature of pertussis, while also highlighting potential limitations in the geographic and demographic representativeness of the sample. This study offers a valuable baseline for future research in Latin America and reinforces the need to strengthen diagnostic capacity to detect coinfections, particularly in epidemic or pandemic contexts.

## INTRODUCTION

Pertussis is a vaccine-preventable highly contagious respiratory disease caused by *Bordetella pertussis*. Since 1978, Colombia has included the diphtheria, tetanus, and pertussis vaccine in the Expanded Program on Immunization, making it mandatory for children under 5 years of age. The current vaccination schedule consists of the first three doses with the pentavalent vaccine (diphtheria, pertussis, tetanus, hepatitis B, and *Haemophilus influenzae* type b [Hib]) administered at 2, 4, and 6 months of age, followed by two booster doses with the Diphtheria, Tetanus, and Pertussis (DPT) vaccine at 18 months and 5 years of age. In addition, since 2014, a booster dose of tetanus**,** diphtheria, acellular pertussis (Tdap) has been administered during the third trimester of pregnancy. The DTP3 vaccine coverage in Colombia was 86.5% in 2021 and 86.96% in 2022.

Clinical presentations of pertussis typically include prolonged cough, with episodes of paroxysmal cough, posttussive vomiting, apnea, or cyanosis. Symptoms can range from a relatively mild cough to severe respiratory illness and complications such as pneumonia, seizures, encephalopathy, respiratory failure, and death (1). While pertussis is caused by *B. pertussis*, other species, such as *Bordetella parapertussis* and *Bordetella holmesii*, are associated with milder respiratory infections in humans presenting as pertussis-like syndrome (2–4).

Pertussis and COVID-19 are respiratory diseases with overlapping clinical manifestations, making differential diagnosis challenging. Since 1997, pertussis surveillance in Colombia has been part of an epidemiological surveillance that integrates risk analysis, case reporting, and laboratory confirmation. The pertussis cases are reported to the National Surveillance System (SIVIGILA) from public and private healthcare institutions; meanwhile, laboratory confirmation of *B. pertussis* is performed by the Microbiology Group at the NRL and by trained network laboratories.

The strict non-pharmaceutical interventions implemented during the COVID-19 pandemic protected the population, especially children and older adults, by reducing virus transmission. However, a notable decline in the incidence of other respiratory pathogens was also observed (5), including *B. pertussis* (6, 7). The global incidence of pertussis dropped significantly during the COVID-19 pandemic, decreasing from 23 cases per 1,000,000 inhabitants in 2019 to 9.9 in 2020 and 4.6 in 2021. However, after the pandemic, an increase in pertussis cases was observed in 2019 to a rise of 23.6 cases per 1,000,000 in 2023. This increase was particularly pronounced in the European region, where the incidence rose from 23.7 to 104 cases per 1,000,000 inhabitants during the same period. Similarly, in the Western Pacific region, cases increased from 6.1 to 26.4 per 1,000,000 (8), highlighting the need for more rigorous epidemiological surveillance due to the risk of complications in patients, coinfections, and mortality cases.

In this context, Colombia experienced a significant decrease in the number of laboratory-confirmed pertussis cases. Compared to 2019 (*n* = 302), confirmed cases decreased by 91.72% (*n* = 25; *P* < 0.05) in 2021 and by 86.75% (*n* = 40; *P* < 0.05) in 2022 (9).

As part of the country’s response to the pandemic and the shifting focus of public health surveillance, the Instituto Nacional de Salud (INS) assumed leadership in coordinating these efforts. During this period, SARS-CoV-2 samples were processed and stored at the NRL of Virology and at some Departmental Public Health Laboratories (DPHL) within the network. Currently, laboratories retain positive SARS-CoV-2 samples stored during this period, providing a valuable resource for research.

The relevance of this study lies in the context of the COVID-19 pandemic, during which the high transmissibility of SARS-CoV-2 highlighted the transmission dynamics of respiratory pathogens. In this scenario, it was considered pertinent to investigate the circulation of *Bordetella pertussis*, given the marked decline in reported cases observed during that period. Studies on coinfections between viral agents and *B. pertussis* are common, with reported coinfection rates reaching 69.7% (10), but, given the limited national and international data on COVID-19 and pertussis coinfections, as well as the availability of relevant samples for both pathogens, this study aimed to assess the positivity of *Bordetella* spp. coinfections in SARS-CoV-2-positive samples from laboratory surveillance at the INS in Colombia during 2021–2022.

## MATERIALS AND METHODS

### Sample selection

Of the samples available (*n* = 12,881), a simple random sample was carried out from which 1,102 (8.6%) samples were selected. Nasopharyngeal samples confirmed as SARS-CoV-2 positive by real-time reverse transcription PCR (11) were analyzed from the period January 2021 to December 2022. All samples were analyzed in the NRL’s Microbiology Group.

### Molecular testing

**Fig 1:**
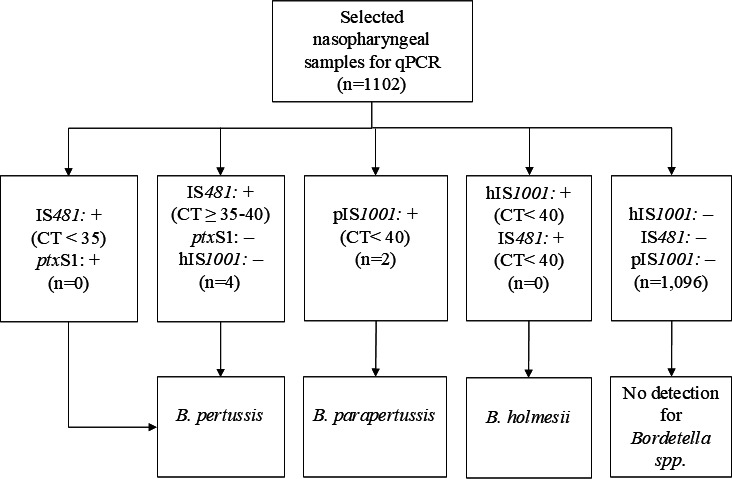
Interpretation of real-time PCR algorithm. IS*481*: multicopy insertion sequence (IS) IS481 target pIS*1001: B. parapertussis* target hIS*1001: B. holmesii* target *ptx*S1: pertussis toxin subunit S1 target

All samples were stored at −80°C until analysis. Bacterial nucleic acids were extracted from 200 µL of nasopharyngeal samples using an automated method with the MagMAX Viral/Pathogen Nucleic Acid Isolation Kit (Thermo Fisher Scientific, USA) on the KingFisher Flex system (Thermo Fisher Scientific, USA). Real-time PCR (qPCR) testing was duplicated to detect *Bordetella* species. DNA eluates were analyzed using a multitarget real-time PCR (qPCR) assay to identify the insertion sequences IS*481*, pIS*1001*, and hIS*1001*, along with a qPCR assay for *RNase* P as a human internal control. This was followed by a confirmatory singleplex assay targeting *ptxS1* (12). The PCR algorithm interpretation is illustrated in Fig. 1. A PCR result was considered negative if the cycle threshold (CT) value was ≥40.

### Data collection

The sociodemographic, epidemiological, and clinical data of the analyzed samples were obtained by cross-referencing the variables recorded in SIVIGILA with the laboratory databases. The records from SIVIGILA and the Virology Group were anonymized and Protected Health Information, ensuring security and confidentiality in their management and analysis.

### Data analysis

Descriptive and bivariate analyses of sociodemographic characteristics, symptoms, and laboratory data were conducted for patients with and without coinfections. To compare the mean duration of clinical course between patients with SARS-CoV-2 infection alone and those with coinfections, a one-way analysis of variance (ANOVA) was performed. Data were analyzed by Epi Info 7 (13) and Microsoft Excel software, and *P* values < 0.05 were considered statistically significant.

To calculate positivity, the number of identified coinfection cases between COVID-19 and pertussis was used as the numerator, and the number of COVID-19-positive samples tested for *Bordetella pertussis* as the denominator. Positivity was expressed as a percentage by applying a multiplication factor of 100. This approach was applied both to estimate annual positivity (2021–2022) and to perform a stratified analysis by departments where coinfection was detected.

To estimate coinfection rates, the exact Clopper-Pearson method was used to calculate confidence intervals for binomial proportions. This method was chosen for its suitability in providing accurate interval estimates, particularly when sample sizes are small or observed proportions are near 0 or 1. Confidence intervals were computed using the OpenEpi software (https://www.openepi.com/BriefDoc/Licensing.htm).

## RESULTS

Of all the samples positive for SARS-CoV-2, 53.45% were female. Of all patients, 50.72% reported cough as the most frequent symptom of COVID-19. Records of pertussis symptoms were not available. The ages of all patients ranged from <1 to 80 years, but the most represented age group (77.58%) was between 20 and 69 years.

Of the samples analyzed, six (0.54%) tested positive for *Bordetella* spp. Among these, four (66.67%) were coinfected with *Bordetella pertussis*, and two (33.33%) with *Bordetella parapertussis*. Among these coinfections, 16.66% (1/6) were Indigenous and 33.3% (2/6) were Black race. The ages of these coinfections range from 20 to 70 years old, but the highest number of coinfections was identified in the age group 20–29 years. A significant difference in the prevalence of fatigue was observed between coinfected patients and those with only COVID-19 (*P* < 0.05), while 75% of coinfected patients reported fatigue, only 16.51% of patients with COVID-19 alone experienced this symptom. Table 1 details other symptoms registered in SARS-CoV-2 patients with and without coinfections.

**TABLE 1:** Characteristics of SARS-CoV-2 patients with *Bordetella* spp. coinfection status, Colombia, 2021-2022

	Coinfection status			Analytic sample, *n* (%)
	SARS-CoV-2 with *B. pertussis*, n (%)	SARS-CoV-2 with *B. parapertussis*, n (%)	SARS-CoV-2 only, n (%)	
Total	*n* = 4	*n* = 2	*n* = 1,096	*n* = 1,102
Age group (years)				
<1	0 (0.00)	0 (0.00)	31 (2.83)	31 (2.81)
1–4	0 (0.00)	0 (0.00)	45 (4.11)	45 (4.08)
5–9	0 (0.00)	0 (0.00)	20 (1.82)	20 (1.81)
10–19	0 (0.00)	0 (0.00)	75 (6.84)	75 (6.81)
20–29	2 (50.00)	1 (50.00)	219 (19.98)	222 (20.15)
30–39	1 (25.00)	0 (0.00)	255 (23.27)	256 (23.23)
40–49	0 (0.00)	0 (0.00)	154 (14.05)	154 (13.97)
50–59	1 (25.00)	0 (0.00)	137 (12.50)	138 (12.52)
60–69	0 (0.00)	0 (0.00)	85 (7.76)	85 (7.71)
>70	0 (0.00)	1 (50.00)	75 (6.84)	76 (6.90)
Sex				
Female	1 (25.00)	1 (50.00)	587 (53.56)	589 (53.45)
Male	3 (75.00)	1 (50.00)	509 (46.44)	513 (46.55)
Department of notification				
Amazonas	0 (0.00)	1 (50.00)	157 (14.32)	158 (14.34)
Atlántico	0 (0.00)	0 (0.00)	15 (1.37)	15 (1.36)
Bogotá D.C	0 (0.00)	0 (0.00)	19 (1.73)	19 (1.72)
Bolívar	0 (0.00)	0 (0.00)	14 (1.28)	14 (1.27)
Caldas	0 (0.00)	0 (0.00)	88 (8.03)	88 (7.99)
Caquetá	0 (0.00)	0 (0.00)	22 (2.01)	22 (2.00)
Casanare	0 (0.00)	0 (0.00)	4 (0.36)	4 (0.36)
Cauca	0 (0.00)	0 (0.00)	3 (0.27)	3 (0.27)
Cesar	0 (0.00)	0 (0.00)	1 (0.09)	1 (0.09)
Chocó	1 (25.00)	1 (50.00)	94 (8.58)	96 (8.71)
Córdoba	0 (0.00)	0 (0.00)	4 (0.36)	4 (0.36)
Cundinamarca	0 (0.00)	0 (0.00)	1 (0.09)	1 (0.09)
Guainía	0 (0.00)	0 (0.00)	19 (1.73)	19 (1.72)
Guaviare	1 (25.00)^*a*^	0 (0.00)	57 (5.20)	58 (5.26)
La Guajira	0 (0.00)	0 (0.00)	157 (14.32)	157 (14.25)
Magdalena	1 (25.00)	0 (0.00)	97 (8.85)	98 (8.89)
Nariño	0 (0.00)	0 (0.00)	21 (1.92)	21 (1.91)
Norte Santander	1 (25.00)	0 (0.00)	65 (5.93)	66 (5.99)
Putumayo	0 (0.00)	0 (0.00)	6 (0.55)	6 (0.54)
San Andres	0 (0.00)	0 (0.00)	57 (5.20)	57 (5.17)
Sucre	0 (0.00)	0 (0.00)	9 (0.82)	9 (0.82)
Tolima	0 (0.00)	0 (0.00)	36 (3.28)	36 (3.27)
Valle	0 (0.00)	0 (0.00)	10 (0.91)	10 (0.91)
Vaupés	0 (0.00)	0 (0.00)	26 (2.37)	26 (2.36)
Vichada	0 (0.00)	0 (0.00)	114 (10.40)	114 (10.34)
Race				
Indigenous	1 (25)	0	64 (5.83)	65 (5.89)
Raizal	0 (0.00)	0 (0.00)	1 (0.09)	1 (0.09)
Black	1 (25)	1 (50)	78 (7.11)	80 (7.25)
Other	2 (50)	1 (50)	904 (82.48)	907 (82.3)
No data	0 (0.00)	0 (0.00)	49 (4.47)	49 (4.44)
Symptoms				
Cough	2 (50)	1 (50)	556 (50.72)	559 (50.72)
Fever	2 (50)	1 (50)	381 (31.76)	384 (34.84)
Sore throat	0 (0.00)	0 (0.00)	158 (14.41)	158 (14.34)
Dyspnea, Shortness of breath	0 (0.00)	0 (0.00)	147 (13.41)	147 (13.34)
Fatigue	3 (75)^*a*^	0 (0.00)	179 (16.32)	182 (16.51)
Rhinorrhea	0 (0.00)	0 (0.00)	136 (12.40)	136 (12.34)
Conjunctivitis	0 (0.00)	0 (0.00)	14 (1.27)	14 (1.27)
Headache	1 (25)	1 (50)	226 (20.62)	228 (20.69)
Diarrhea	0 (0.00)	0 (0.00)	77 (7.02)	77 (6.99)
No symptoms/no data	0 (0.00)	0 (0.00)	349 (31.84)	349 (31.6)
Severe complications				
Hospitalized	0 (0.00)	0 (0.00)	107 (9.71)	107 (9.71)
Death	0 (0.00)	0 (0.00)	35 (3.18)	35 (3.18)

^*a*^ *P* < 0.05.

A significant difference (*P* < 0.05) was observed in the mean duration of clinical course between patients with only SARS-CoV-2 (x̄ = 5.84 ± 23.63) and those with coinfections in 2021 (x̄ = 8.16 ± 5.42). Furthermore, there were no deaths or hospitalizations among patients with coinfections.

### Positivity of coinfections

The coinfection positivity of SARS-CoV-2 with *Bordetella* spp. in 2021 was 0.75% cases (four cases), while in 2022, it was 0.34% cases (two cases). In both years, the estimate of coinfection does not exceed the positivity of 2.0% (Table 2).

**TABLE 2:** Positivity of SARS-CoV-2 with *Bordetella* spp. coinfection in Colombia, 2021–2022^*a*^

Year	SARS-CoV-2 collected samples	SARS-CoV-2 with *Bordetella* coinfection	Positivity of SARS-CoV-2 with *Bordetella* coinfection (%)	Lower limit (%)	Upper limit (%)
2021	528	4	0.75	0.20	1.92
2022	574	2	0.34	0.04	1.25

^*a*^ The positivity estimation of SARS-CoV-2 with *Bordetella* spp. coinfection was obtained using a multiplication coefficient of 100, applying the confidence interval according to Fisher's exact method (Clopper-Pearson).

In 2021, positivity was confirmed in the departments of Chocó, Norte de Santander, and Guaviare. The highest positivity was identified in Guaviare, with 5.26% (1/19). The departments of Chocó and Guaviare exceeded the positivity of 1.0% in the estimation of coinfection between both diseases (Table 3).

**TABLE 3:** Incidence of SARS-CoV-2 with *Bordetella* spp. coinfection by territorial entity, 2021–2022, Colombia^*a*^

Year	Territorial entity	SARS-CoV-2 collected samples	SARS-CoV-2 with *Bordetella* coinfection	Positivity of SARS-CoV-2 with *Bordetella* coinfection (%)	Lower limit (%)	Upper limit (%)
2021	Chocó	95	2	2.08	0.25	7.32
	Norte de Santander	63	1	1.58	0.03	8.53
	Guaviare	19	1	5.26	0.13	26.03
2022	Magdalena	10	1	10	0.25	44.5
	Amazonas	91	1	1.09	0.02	5.97

^*a*^ The positivity estimation of SARS-CoV-2 with *Bordetella* spp. coinfection was obtained using a multiplication coefficient of 100, applying the confidence interval according to Fisher's exact method (Clopper-Pearson).

For 2022, coinfection was identified in two departments: Magdalena and Amazonas. The highest positivity was observed in Magdalena, with 10.0% (1/10). The estimation of coinfection in Amazonas was 1.09% (1/91) (Table 3).

## DISCUSSION

This study identified laboratory-confirmed coinfections with *Bordetella* spp*.* in SARS-CoV-2 patients in Colombia during 2021–2022. Our findings are consistent with those of another study (14), which also reported a low proportion (0.78%) of SARS-CoV-2/*Bordetella* spp. coinfections. Although the number of coinfections identified was low, this trend was expected due to the non-pharmacological measures adopted during the pandemic, such as social isolation, mask usage, and increased hygiene awareness, which contributed to a decrease in pertussis incidence (15–19). This reduction would limit the opportunities for coinfections, even if SARS-CoV-2 were widely circulating.

Coinfections involving not only *B. pertussis* but also *B. parapertussis* were identified. While *B. parapertussis* is not a major cause of mortality, sporadic cases unrelated to pertussis outbreaks have been reported (20–22). Notably, the diphtheria-tetanus-pertussis (DTP) vaccine does not provide effective protection against *B. parapertussis* (23), and some cases manifest with like-pertussis symptoms. Given this, testing for both *B. pertussis* and *B. parapertussis* in patients with whooping cough symptoms is crucial. In response to this need, the Microbiology Group has been monitoring *B. parapertussis* using qPCR since 2014 (24).

Despite this, the vaccination history of the patients in the samples analyzed in this study was not available. However, 91.29% of the population was over 10 years old, likely lacking protection due to the waning immunity conferred by the pertussis vaccine (25).

In Colombia, the only documented report of SARS-CoV-2 and *Bordetella* spp*.* coinfections was reviewed by the INS. In 2020, four cases of coinfection between pertussis and COVID-19 were confirmed, of which 50% (2/4) affected children under 1 year of age. The predominant symptoms were cough and paroxysmal cough, with an average duration of 11 days, while 25% (1/4) presented complications such as pneumonia (26). Our results suggest that patients with coinfections exhibited more clinical manifestations, such as fatigue, compared to those with SARS-CoV-2 alone.

In 2021, the INS notified four possible coinfections (4/75) involving *B. pertussis* and other respiratory viruses, one of which was a coinfection with SARS-CoV-2 (1/75). This coinfection occurred in the Department of Choco, where a large-scale pertussis outbreak affected an indigenous community (27). In 2022, a similar outbreak was reported in the Indigenous population of the Sierra Nevada de Santa Marta (28).

Our findings suggest that SARS-CoV-2 patients with coinfections were more frequently Indigenous or had epidemiological links to Indigenous communities. These populations are particularly vulnerable, as many are not enrolled in the Social Health Security System and often lack complete vaccination schedules (27). In Colombia, pertussis has predominantly affected Indigenous and rural communities. However, it is important to highlight that the samples analyzed in this study were drawn from an urban population, which may partially account for the low observed coinfection rate.

In addition, coinfections with *Bordetella* spp. were identified in the departments of Guaviare and Amazonas. However, the number of reported pertussis cases in these regions was very low (9), indicating an epidemiological silence surveillance system.

The coinfections were more frequent in young adult patients. In the study by Roh et al. (29), the median age of the SARS-CoV-2 patients with bacterial coinfections was reported as 32.0 ± 6.0 years, implying that young adults may be more susceptible to respiratory pathogen coinfections.

Some patients with SARS-CoV-2, only in 2021, reported an unusually prolonged duration of symptoms. This suggests that they may have coinfections with other respiratory pathogens. Chen et al. (30) summarized the most common microbial coinfections with SARS-CoV-2, highlighting respiratory viruses such as coronavirus (non-COVID-19) (2.1%), Entero/rhinovirus (hRV) (6.9%), influenza A (4.57%), human metapneumovirus (hMPV) (1.98%), and respiratory syncytial virus (RSV) (3.46%). Bacterial coinfections accounted for 4.97% and included *Acinetobacter baumannii*, *Actinomyces* spp., and *Klebsiella pneumoniae*. Fungal coinfections were observed in 3.16% of cases, involving *Legionella pneumophila*, *Rothia* spp., *Streptococcus* spp., *Veillonella* spp., *Aspergillus* spp., and *Candida* spp.

On the other hand, several studies reported coinfections in pertussis patients with other respiratory viruses. Ferronato et al*.* (31) identified coinfections in 7% of cases, with 4.9% specifically involving respiratory syncytial virus (RSV). Scutari et al. (10) found a high coinfection rate (69.76%) with respiratory viruses, including Human Rhinovirus/Enterovirus (7/43), hMPV (3/43), Parainfluenza virus type 3 (PIV3) (1/43), coronavirus OC43 (1/43), and RSV (1/43), among others.

In addition, the INS documented a pertussis outbreak in 2024 among an Indigenous population in Urrao and Betulia, Antioquia, confirming 12 cases (32). Among these, coinfections with other respiratory agents were identified, including influenza A(H3) (25%), adenovirus (66.6%), and RSV (58.33%). This finding reinforces the hypothesis of potential interactions between pertussis and various respiratory pathogens and underscores the occurrence of such interactions across different geographic regions and population contexts.

In the Americas, the incidence of pertussis declined significantly between 2020 and 2023, dropping from 20.1 to 6.1 cases per 1,000,000 inhabitants. This reduction was primarily due to the redirection of surveillance efforts toward SARS-CoV-2, which temporarily affected the detection and reporting of *Bordetella pertussis* cases. However, as of 2024, incidence rates are stabilizing and returning to pre-pandemic levels, reflecting the renewed focus on pertussis surveillance and its natural epidemiological trends. Several countries, including the United States, Mexico, Peru, and Brazil, are experiencing a resurgence, indicating that *B. pertussis* circulation was low during the analyzed period but is now reestablishing itself (33).

In Colombia, the lower coinfection rate observed during this period can also be attributed to the natural cyclical behavior of *B. pertussis*, which follows a 4-year pattern of fluctuating case numbers (34). In addition, protective measures implemented to mitigate the spread of SARS-CoV-2, such as mask-wearing, social distancing, and restricted mobility, likely contributed to reducing the transmission of *B. pertussis* and other respiratory pathogens.

The study also had some limitations. First, there was a low inclusion of samples from Antioquia, Bogotá D.C., and the Cundinamarca department that reported the highest number of pertussis cases. This may have impacted the representativeness by department of our findings. Second, there was a lack of information on symptoms or medical records to associate pertussis symptoms in the analysis.

These results serve as a reference framework for the study of COVID-19 and pertussis coinfection in Latin America and are the first to confirm coinfections between SARS-CoV-2 with *B. pertussis* and *B. parapertussis* in Colombia.

A deeper follow-up through studies or syndromic surveillance is essential for detecting multiple pathogens and gaining a better understanding of coinfection dynamics. This approach would help identify the most affected populations and assess the potential public health impact. The need for such surveillance is particularly relevant for children under 5 and adults over 60, who are more vulnerable to severe complications. Due to their developing or aging immune systems, these populations face a higher risk of adverse outcomes, highlighting the importance of tailored prevention and treatment strategies to address their specific needs.

## AFTER EPUB

*[This article was published on 12 September 2025 with errors in Acknowledgments. The Acknowledgments were corrected in the current version, posted on 17 September 2025.]*
